# Stem Type as an Independent Correlate of Functional Outcome Following Total Hip Arthroplasty: A Comparative Study of Short-Stem and Standard-Stem Femoral Implants

**DOI:** 10.3390/jfb17100499

**Published:** 2026-10-02

**Authors:** Michał Urbaniak, Joanna Rywaniak, Dominika Szczerbiec, Mateusz M. Urbaniak, Wojciech Dąbrowski, Ireneusz Urbaniak, Marek Synder

**Affiliations:** 1 Department of Orthopedics and Traumatology, Ludwik Perzyna Provincial Integrated Hospital in Kalisz, Poznańska 79, 62-800 Kalisz, Poland; 2 Department of Immunology and Infectious Biology, Faculty of Biology and Environmental Protection, University of Lodz, Banacha 12/16, 90-237 Lodz, Poland; joanna.rywaniak@biol.uni.lodz.pl; 3 Faculty of Medicine and Health Science, University of Kalisz, Plac Wojciecha Bogusławskiego 2, 62-800 Kalisz, Poland; mateusz.urbaniak@uniwersytetkaliski.edu.pl (M.M.U.); 36389@uniwersytetkaliski.edu.pl (W.D.); i.urbaniak@uniwersytetkaliski.edu.pl (I.U.); 4 Medical Center Ksiezy Mlyn, 90-338 Lodz, Poland; msynder@pro.onet.pl

**Keywords:** total hip arthroplasty, conventional stem, short stem

## Abstract

Idiopathic hip osteoarthritis is a major cause of disability worldwide, and total hip arthroplasty (THA) is the standard treatment for advanced disease; however, the independent effect of femoral stem design on functional outcomes remains unclear. This retrospective cohort study compared clinical, biochemical, and radiographic outcomes between short-stem and standard-stem hip prostheses and identified predictors of superior postoperative function. A total of 70 patients undergoing primary THA for idiopathic coxarthrosis were divided into two equal groups based on implant type. Functional outcomes were assessed using the Harris Hip Score (HHS) and WOMAC preoperatively and 6 weeks postoperatively, along with perioperative laboratory parameters and radiographic evaluation. Both groups showed significant postoperative improvements in HHS and WOMAC scores, with no significant difference in HHS between groups. The short-stem group achieved significantly better WOMAC total and function scores, whereas the standard-stem group showed slightly better stiffness outcomes. No differences were found in postoperative inflammatory response or radiographic alignment. Multivariable analysis identified stem type as the only variable significantly and independently associated with superior functional outcome, with short-stem implants associated with higher odds of favorable results. This association was robust to adjustment for baseline functional status in sensitivity analysis. These findings suggest that both designs are effective, while short-stem prostheses are associated with better early patient-reported functional outcomes at 6 weeks after THA.

## 1. Introduction

Idiopathic osteoarthritis of the hip joint is a growing medical and economic problem in aging societies. Osteoarthritis is the most common joint disease, affecting more than 240 million people worldwide and representing approximately 7.6% of the global population, with a projected increase of 60–100% by 2050 [1,2]. It occurs primarily in middle-aged and older individuals [3] and is estimated to be the second most common cause of disability in Europe after cardiovascular diseases. In Poland, approximately 8 million people suffer from osteoarthritis, with around 40% affected by hip OA [4]. Longer lifespans, obesity, increased physical activity, and the rising incidence of metabolic and inflammatory disorders contributed to a marked increase in degenerative joint diseases in the second half of the 20th century [5].

Osteoarthritis is caused by the loss of collagen and proteoglycans in articular cartilage, leading to deterioration of its biomechanical properties. This process is followed by chondrocyte damage and the release of cytokines and other proinflammatory mediators [6,7], resulting in progressive cartilage degeneration, surface irregularities, and reduced cartilage thickness [3].

Osteoarthritis is not merely a process of mechanical wear and damage to the articular surfaces, but rather a complex inflammatory metabolic disorder involving the activity of pro-inflammatory cytokines, including interleukins (IL)-1β, IL-6, IL-17, and tumor necrosis factor-α (TNF-α). These mediators stimulate excessive secretion of metalloproteinases by chondrocytes, ultimately leading to the degradation of type II collagen [8,9]. Elevated levels of these cytokines in the joints also contribute to the inhibition of extracellular matrix synthesis, chondrocyte apoptosis, and the induction of pain mediators, including cyclooxygenase-2 (COX-2) and prostaglandin E2 (PGE2) [7,9,10].

Clinically, this manifests as joint pain, limping, and limited range of motion; in advanced stages, limb shortening and characteristic positioning of the hip joint in flexion, external rotation, and adduction may occur [11,12]. In the final stages, the joint may become stiff [5], significantly reducing quality of life and limiting physical and social activity [13].

Among the various etiologies of hip joint degeneration leading to total hip arthroplasty, idiopathic osteoarthritis remains the most common cause [14]. Risk factors include occupations requiring heavy physical labor, such as farming or jobs involving lifting, as well as high-level sports activity [15]. Initial management of early-stage disease is conservative and includes lifestyle modification, physical therapy, and the use of assistive devices such as crutches [16]. Pharmacological treatment primarily involves nonsteroidal anti-inflammatory drugs and intra-articular steroid injections, while supplements such as chondroitin sulfate or glucosamine are also used despite limited evidence supporting their efficacy [17].

Since the 1960s, total hip arthroplasty has undergone significant development and is now widely recognized as an effective treatment for advanced degenerative hip disease; it was notably described as the “operation of the century” in *The Lancet* [18,19]. Currently, most implants are cementless and differ in shape, surface characteristics, fixation methods, and stem geometry [20]. One of the key classification criteria for hip prostheses is stem design, which includes standard and short stems. The primary differences lie in stem length, shape, and the extent of bone resection required. Metaphyseal (short-stem) prostheses preserve more bone compared to standard stems [21]. In recent years, the use of short stems has increased [22], partly due to reports of reduced mid-thigh pain in physically active patients receiving metaphyseal implants [23].

The clinical success of total hip arthroplasty is closely associated with the quality of osseointegration and the maintenance of physiological stress distribution within the peri-implant bone tissue. In an optimally fitted prosthesis, osseointegration proceeds effectively through the uniform transfer of mechanical loads to the surrounding bone, in accordance with Wolff’s law [24]. Incorrect implant fitting, including inappropriate prosthesis size, length, or valgus and varus alignment, may result in implant micromotion. This phenomenon compromises primary stability and increases the risk of early prosthetic loosening [25,26]. Moreover, abnormal load distribution enhances friction at the bone–implant interface, thereby promoting the formation of microcracks and the release of prosthetic wear particles [27]. These particles activate osteoclasts to secrete pro-inflammatory mediators, including IL-1, IL-6, TNF-α, and RANKL, which stimulate osteoclastogenesis and consequently increase the risk of aseptic periprosthetic osteolysis [28].

Direct comparative evidence regarding functional outcomes between short and conventional stems in homogeneous patient populations remains limited, and the independent effect of stem type on postoperative functional performance has not yet been fully established. Hip replacement in advanced degenerative disease relieves pain, restores limb function, and allows patients to return to their expected activities, including professional work. The aim of this study was twofold: (1) to compare functional and laboratory outcomes between patients receiving short-stem and standard-stem hip prostheses, and (2) to identify independent predictors of favorable functional outcomes following total hip arthroplasty in patients with idiopathic hip osteoarthritis.

## 2. Materials and Methods

### 2.1. Characteristics of Study Group and Implants

The retrospective cohort study included patients treated for idiopathic hip osteoarthritis at the Department of Orthopedics and Traumatology, Ludwik Perzyna Provincial Integrated Hospital in Kalisz, Poland, between 2012 and 2015. The study was conducted in accordance with the Declaration of Helsinki, and the protocol was approved by the Ethics Committee of Medical University in Lodz (RNN/66/11/KE).

In total, 70 patients were included in the analysis, comprising 33 men and 37 women. Patients were divided into two groups according to the femoral stem used. The inclusion criteria comprised age ≥ 18 years, qualification for elective hip joint arthroplasty due to idiopathic coxarthrosis, and the availability of a complete set of biochemical laboratory results. Patients with cancer, pregnant women, individuals with bone or skin infections at the surgical site, and those with coxarthrosis of non-idiopathic origin were excluded.

Group 1 (standard-stem): 35 patients, including 13 men and 22 women, aged 54 to 74 years at the time of surgery. Two complications were observed: one case of peroneal nerve neuropraxia and one case of periprosthetic fracture.

Group 2 (short-stem): 35 patients, including 20 men and 15 women, aged 48 to 80 years at the time of surgery. One postoperative complication was recorded: a dislocation on postoperative day 11. No further complications were reported during follow-up.

All surgical procedures were primary cementless THAs performed by a single experienced orthopedic surgeon, using a standardized posterolateral approach to the hip in both study groups. The choice of femoral stem was made by the operating surgeon based on preoperative radiographic assessment and intraoperative evaluation of the femoral anatomy and fixation conditions. The decision took into consideration femoral canal morphology, metaphyseal bone stock, and the anticipated ability to achieve adequate fixation and appropriate stem engagement. Stem allocation was therefore based on individual clinical and anatomical assessment rather than randomization. No formal written allocation algorithm based on patient age or Dorr classification was used.

Both groups were managed according to the same institutional perioperative pathway, including comparable anesthetic and transfusion protocols and routine postoperative laboratory monitoring. Postoperative care followed a uniform departmental protocol based on early mobilization and physiotherapist-supervised gait training. Weight-bearing was prescribed according to intraoperative implant stability and departmental standards rather than implant type alone.

Two types of cementless femoral stems were used: standard-stem and short-stem implants. These implants differ primarily in stem length: the standard-stem implant represents a conventional full-length design, whereas the short-stem prosthesis has a shorter, bone-preserving configuration that may offer advantages in the event of future revision surgery [29]. The clinical follow-up period ranged from 3 to 6 years and was used to monitor implant-related outcomes, including implant stability, revisions, and complications.

Two cementless femoral stem designs were used in this study: a standard-length stem and a short bone-preserving stem. The standard stem was characterized by a straight, proximally tapered geometry with trapezoidal proximal and quadrangular distal cross-sections, designed to provide axial and rotational stability while minimizing cortical contact [30]. It featured a full hydroxyapatite-coated surface to promote rapid osseointegration and biological fixation. Neck-shaft angle options included standard/high-offset (135°) and varus configurations (125°), depending on patient anatomy [31]. The short stem was characterized by a curved, metaphyseal-fitting design adapted to the native femoral canal, with a three-dimensional tapered wedge geometry. Its proximal trapezoidal cross-section enhanced rotational stability, while the shortened distal portion preserved femoral bone stock [32]. The stem was manufactured from titanium alloy (Ti-6Al-4V) and featured a porous titanium-coated proximal surface for press-fit fixation and osteointegration, with a roughened distal surface [33]. Multiple neck-shaft angle configurations were available to optimize biomechanical reconstruction and offset restoration [34].

### 2.2. Clinical Evaluation

Data contained in the patients’ medical records, including medical histories, as well as general, departmental, and operative records were analyzed to assess the indications for arthroplasty, the course of the surgery, and the postoperative period. Clinical evaluation was conducted using the Harris Hip Score (HHS) [35] and the Western Ontario and McMaster Universities Osteoarthritis Index (WOMAC) [36], both widely applied in orthopedic research and practice. Patients completed the questionnaires preoperatively and again 6 weeks postoperatively. HHS and WOMAC were not systematically reassessed during the subsequent 3–6-year clinical follow-up; therefore, these measures were used exclusively to evaluate early postoperative functional recovery. The HHS is a validated instrument for assessing hip joint disorders, including outcomes following total hip arthroplasty. It incorporates both subjective measures (pain and function) and objective measures (range of motion and deformity) [37]. The scale assesses joint mobility, deformities, functioning, and pain, with a maximum score of 100 points [38].

WOMAC is a proven, multilayered tool for evaluating impairment in patients with hip osteoarthritis, with scores ranging from 0 (best) to 96 (worst). The WOMAC evaluates treatment outcomes based on patient-reported pain, stiffness, and functional impairment of the hip. It comprises 24 items: 5 addressing pain, 2 addressing stiffness, and 17 addressing physical function [3,4,39]. It is widely used for assessing hip and knee osteoarthritis as well as treatment outcomes [40].

### 2.3. Biochemical Evaluation

Serum biochemical parameters, including C-reactive protein (CRP), hemoglobin (HGB), and leukocyte count (LEU), were assessed 24 h preoperatively (pre-op) and 48–72 h postoperatively (post-op) in all patients undergoing hip arthroplasty. Blood samples were collected as part of routine perioperative laboratory evaluation performed in accordance with standard hospital protocols. Biochemical analyses were conducted using the Beckman Coulter DxC 700 AU (Beckman Coulter Inc., Brea, CA, USA) automated chemistry analyzer.

### 2.4. Radiographic Imaging and Measurements

Postoperative outcomes following total hip arthroplasty were evaluated using anteroposterior (AP) radiographs including the proximal one-third of the femur, obtained in the supine position, to assess implant positioning, stability, and the presence of potential complications. All radiographs were acquired using a Siemens Mobilett XP (Siemens Healthcare GmbH, Erlangen, Germany) system with Canon Exposure Display Interface VC10 software (Canon Inc., Tokyo, Japan) at the Department of Diagnostic Imaging, Ludwik Perzyna Provincial Integrated Hospital in Kalisz. Radiographic assessment focused on stem alignment, determined by measuring the angle between the longitudinal axis of the prosthetic stem and the axis of the proximal femoral shaft; based on this measurement, stem positioning was classified as neutral, valgus, or varus. The stem alignment was classified as neutral, varus, or valgus according to literature data [41]. It was considered neutral when the vertical axis of the stem was within 0 to 2° relative to the axis of the femoral shaft. Deviations greater than 2° in the medial direction were classified as varus, while deviations greater than 2° in the lateral direction were classified as valgus. The radiographic assessment in the present study was focused on postoperative stem positioning and alignment. Serial quantitative assessment of periprosthetic bone remodeling, including longitudinal Gruen-zone analysis or bone mineral density measurements, was not included in the study protocol.

### 2.5. Statistical Analysis

Statistical analysis was conducted using Python (v. 3.x) with the following libraries: pandas and numpy for data management, and scipy.stats for inferential statistics.

The normality of the data distribution was assessed using the Shapiro–Wilk test. Due to the non-normal distribution of continuous variables (age, BMI, inflammatory markers, HHS, and WOMAC scores), they were expressed as medians with interquartile ranges (IQR: 25th–75th percentiles). Categorical variables, such as gender, were presented as absolute numbers and percentages.

Differences between the short-stem and standard-stem groups at specific time points were evaluated using the Mann–Whitney U test for continuous variables and the Chi-square test (or Fisher’s exact test where appropriate) for categorical data. Although this retrospective cohort study was not formally pre-registered in a public trial’s registry, the statistical evaluation plan was fully pre-specified in our institutional analytical protocol prior to data evaluation. Fisher’s exact test was specifically applied to evaluate differences in radiographic stem alignment (neutral, varus, or valgus) between the study groups due to the low expected frequencies in certain categories. The effect size for statistically significant between-group differences was quantified using the rank-biserial correlation coefficient (r). The results were interpreted according to Cohen’s criteria: 0.1–0.3 as a small effect, 0.3–0.5 as a medium effect, and ≥0.5 as a large effect. To account for multiple testing across secondary and subscale endpoints, Benjamini–Hochberg false discovery rate (FDR) adjustments were applied where appropriate, and adjusted *p*-values are reported alongside nominal values.

To assess the efficacy of the surgical intervention within each group, preoperative and postoperative values—including HHS, WOMAC scores, and laboratory parameters (CRP, HGB, LEU)—were compared using the Wilcoxon signed-rank test.

The relationship between patient-specific factors (age, BMI) and postoperative clinical scores (WOMAC total and its subscales) was investigated using Spearman’s rank correlation analysis. Furthermore, the impact of radiographic stem positioning (neutral vs. non-neutral alignment) on clinical outcomes (HHS and WOMAC subscales), including potential interaction effects with stem type, was analyzed using the Mann–Whitney U test, with Benjamini–Hochberg FDR adjustments applied to account for multiple comparisons, to identify potential associations between implant orientation and patient-reported stiffness or pain. A multivariable logistic regression model was implemented to identify factors independently associated with a superior early functional outcome. Logistic regression was selected as the primary analytical method given the binary nature of the primary variable of interest (stem type: short-stem vs. standard-stem) and the clinical objective of estimating the odds of achieving a superior outcome associated with each implant type. The dependent variable was defined as a postoperative total WOMAC score at or below 18 points, corresponding to the median value of the entire study cohort (*n* = 70), adopted as a pragmatic threshold in the absence of a universally validated cut-off for a superior functional outcome following total hip arthroplasty. The model included stem type, age, and BMI as independent variables, selected a priori based on clinical relevance; the number of predictors was intentionally limited to reduce the risk of overfitting, in accordance with the available sample size and the events-per-variable (EPV) principle, supported by information criteria (AIC/BIC) evaluation to corroborate the superior simplicity of the primary model. Additionally, to test the robustness of our findings and account for potential baseline functional differences, a sensitivity analysis was conducted by incorporating the preoperative total WOMAC score as an additional covariate in the multivariable logistic regression model. The standard-stem prosthesis was designated as the reference category for the stem type variable. Results are reported as odds ratios (OR) with 95% confidence intervals (CIs).

Statistical significance for all tests was set at a two-tailed *p*-value < 0.05.

## 3. Results

### 3.1. Baseline Characteristics

The two study groups were comparable in terms of age, sex distribution, and BMI, with no statistically significant differences observed between them (Table 1). No statistically significant baseline differences were detected; however, given the limited sample size (*n* = 35 per group), the study was not powered to formally demonstrate group equivalence, and residual imbalance in unmeasured or continuous baseline characteristics cannot be excluded.

### 3.2. Perioperative Laboratory Parameters

Both groups demonstrated the expected postoperative increase in inflammatory markers (C-reactive protein, leukocytes) and a decrease in hemoglobin levels (Table 2). Preoperative CRP was significantly higher in the standard-stem group (*p* = 0.003). This difference may reflect unmeasured patient-related or biological factors; however, the retrospective design of the study precludes determination of its underlying cause. However, no between-group differences were observed in the postoperative inflammatory response (*p* = 0.119), suggesting a comparable systemic reaction to surgery regardless of implant type.

### 3.3. Clinical Outcomes

**Figure 1:**
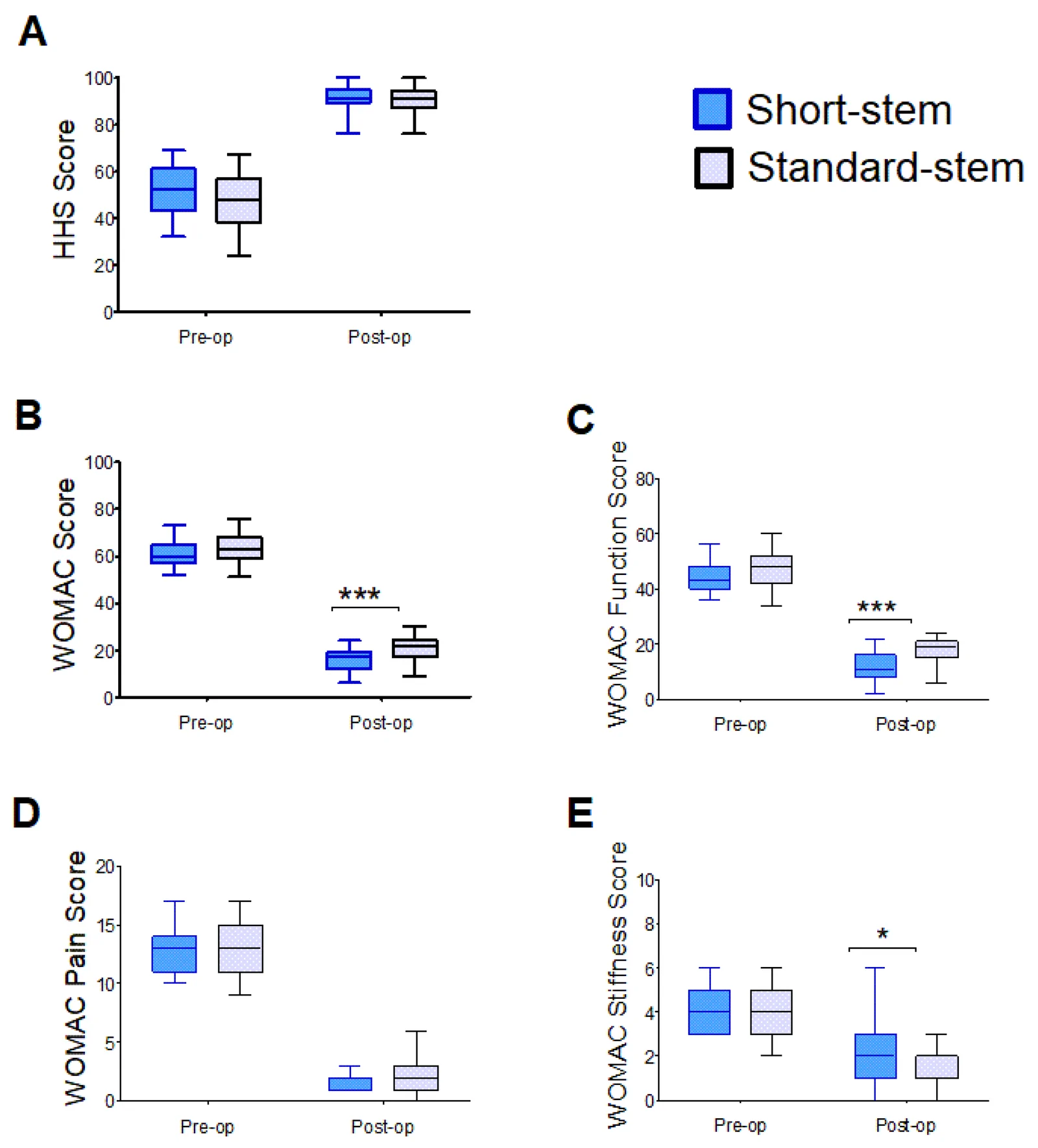
Comparison of clinical outcomes between short-stem and standard-stem study groups. Data are presented as medians (horizontal line), interquartile ranges (box), and 5th–95th percentiles (whiskers). (**A**) Harris Hip Score (HHS) showed no significant differences between groups. (**B**) Total WOMAC score and subscales for (**C**) Function, (**D**) Pain, and (**E**) Stiffness. Statistically significant differences between groups (Mann–Whitney U test) are indicated by asterisks (* *p* < 0.05, *** *p* < 0.001). Lower WOMAC scores represent superior clinical outcomes.

At the 6-week postoperative assessment, both prosthesis types resulted in significant improvements in functional outcomes compared with baseline (Table 3, Figure 1). The Harris Hip Score (HHS) improved substantially in both groups postoperatively, with no significant between-group difference (*p* = 0.426), indicating equivalent overall hip function regardless of stem type.

However, at 6 weeks postoperatively, WOMAC total scores differed significantly between groups (*p* < 0.001; effect size r = 0.45), with the short-stem group achieving lower scores (median 17.0 vs. 22.0 points), corresponding to better early postoperative patient-reported functional outcomes. Subscale analysis revealed that the short-stem group performed significantly better in the Function domain (*p* < 0.001; r = 0.62, large effect), while the standard-stem group demonstrated lower Stiffness scores postoperatively (*p* = 0.013; r = 0.26, small effect). No significant between-group difference was found for the Pain subscale (*p* = 0.056).

### 3.4. Correlation Analysis

Spearman’s rank correlation analysis revealed no significant associations between patient characteristics—age or BMI—and postoperative WOMAC scores (Table 4), suggesting that functional outcomes following hip arthroplasty were independent of patient demographic profile.

### 3.5. Multivariable Logistic Regression

**Figure 2:**
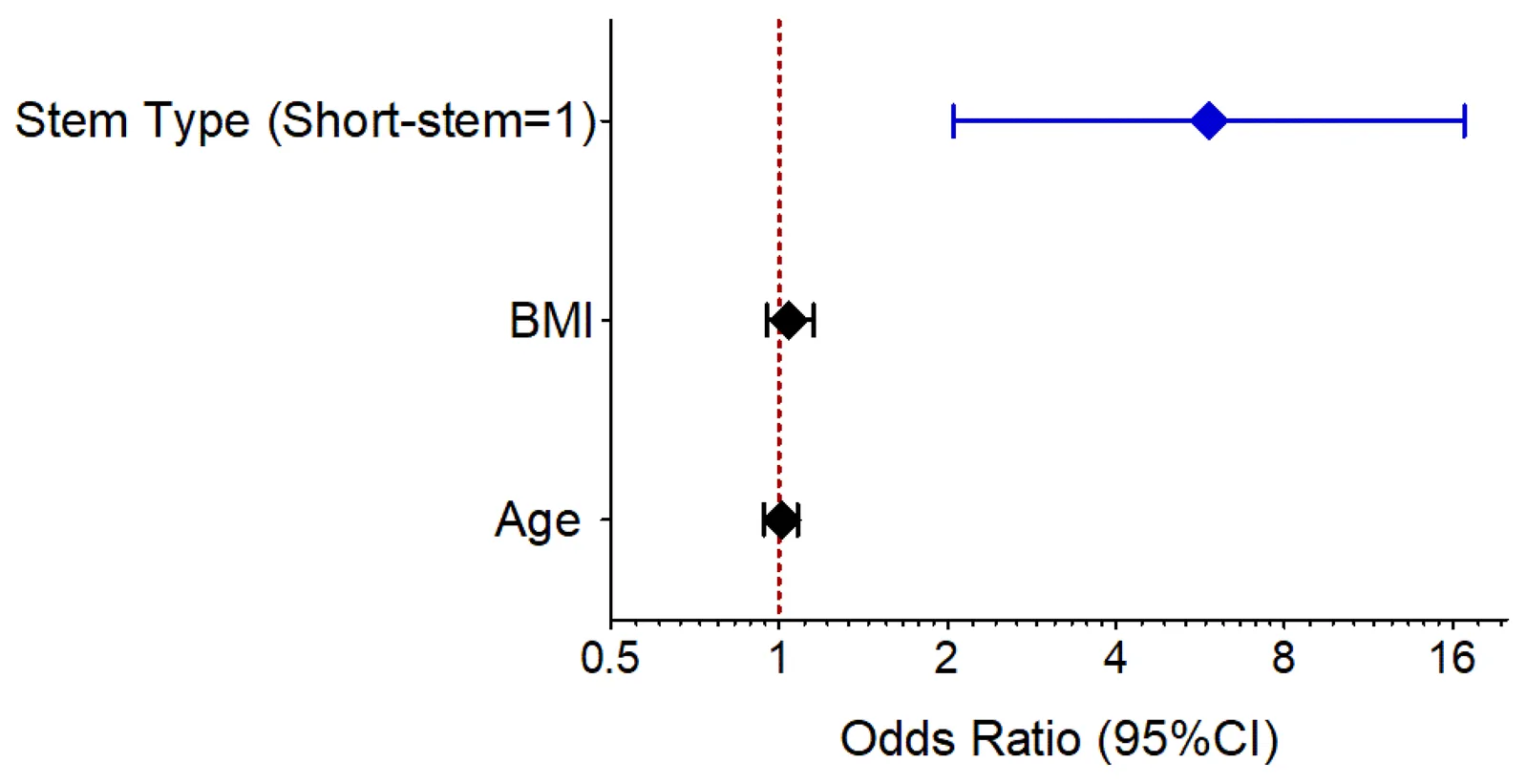
Predictors of superior functional outcome (postoperative WOMAC score ≤ 18 points). Forest plot representing multivariable logistic regression analysis. Results are shown as odds ratios (OR) with 95% confidence intervals (CI). The vertical dashed line at OR = 1.0 represents the null effect. The short-stem type was the only significant independent correlate of a superior outcome (OR = 5.87, *p* = 0.001), while age and BMI did not significantly influence the results.

Multivariable logistic regression identified stem type as the only variable independently associated with the predefined favorable functional outcome, defined as a postoperative WOMAC score at or below the median of 18 points (Table 5, Figure 2). Patients treated with the short-stem prosthesis had nearly six times higher odds of achieving this threshold compared to those receiving the standard-stem implant (OR = 5.87; 95% CI: 2.05–16.80; *p* = 0.001). Neither age (OR = 1.01; *p* = 0.787) nor BMI (OR = 1.04; *p* = 0.395) significantly influenced the likelihood of a superior outcome. To account for potential baseline functional disparities, a sensitivity analysis was subsequently performed incorporating the preoperative total WOMAC score as an additional covariate. In this expanded model, the association between stem type and superior outcome remained essentially unchanged (OR = 5.92; 95% CI: 2.07–16.95; *p* = 0.001), whereas preoperative WOMAC failed to reach statistical significance (OR = 1.00; *p* = 0.942). Furthermore, model evaluation via information criteria demonstrated a slight increase in both Akaike Information Criterion (AIC, from 92.13 to 94.12) and Bayesian Information Criterion (BIC, from 101.12 to 105.36), confirming that the inclusion of the baseline score did not improve overall fit and corroborating the superior simplicity of the primary model. Consequently, these findings indicate that the observed treatment effect is not attributable to the near-significant baseline WOMAC imbalance alone (*p* = 0.065, Table 3). Nonetheless, given the retrospective, non-randomized design of the study, potential residual confounding by unmeasured clinical variables (such as detailed femoral morphology, bone quality, metaphyseal bone stock, or other clinical and anatomical factors influencing implant selection) cannot be entirely ruled out; thus, the designated predictors are interpreted as being independently associated with the outcome rather than as definitive evidence of a direct causal relationship.

### 3.6. Radiographic Evaluation

**Figure 3:**
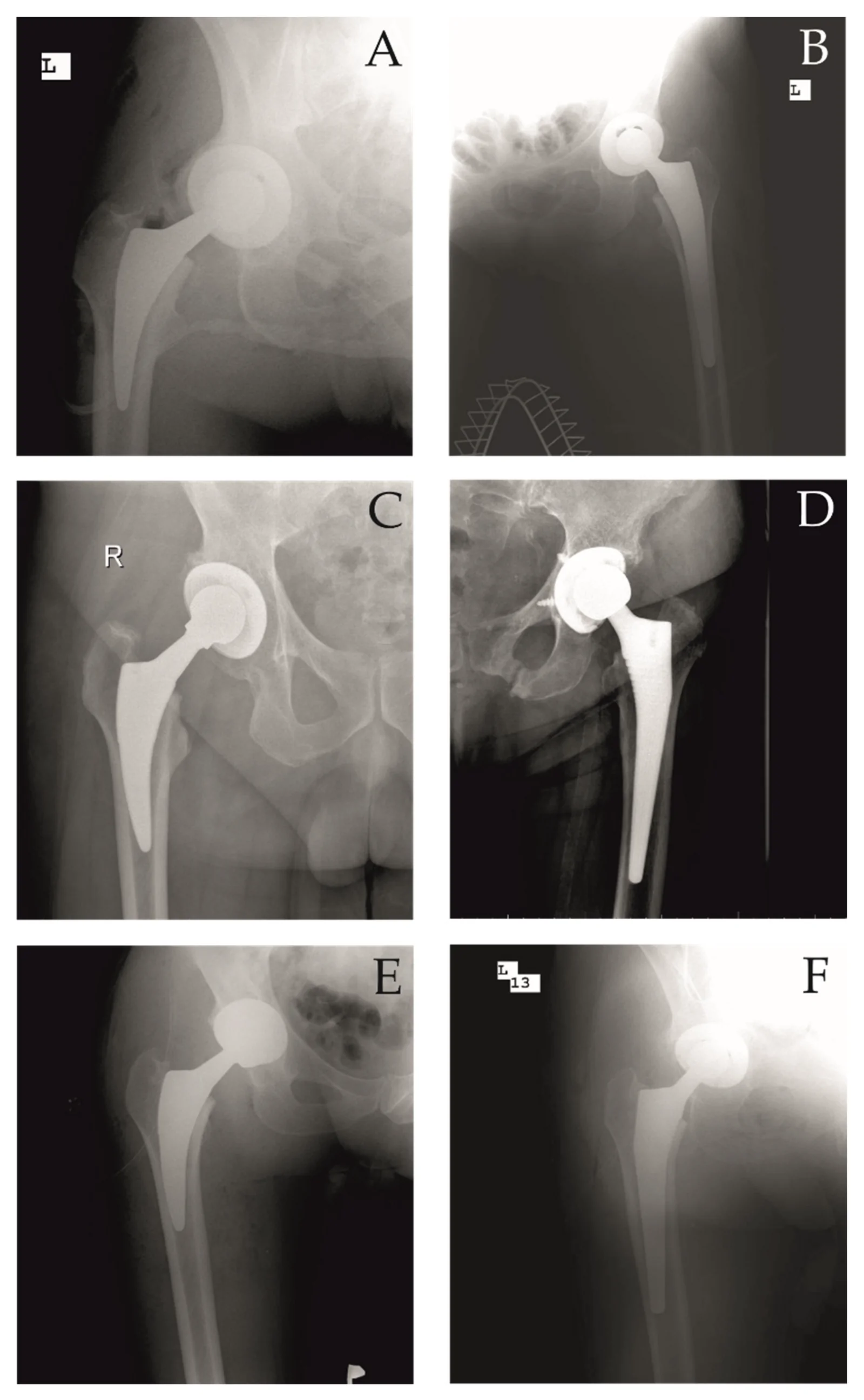
Postoperative radiographs demonstrating neutral positioning of short-stem (**A**) and standard-stem (**B**), valgus positioning of short-stem (**C**) and standard-stem (**D**) and varus positioning of short-stem (**E**) and standard-stem (**F**) hip prostheses.

Radiographs were used to assess the correct implantation of the endoprosthesis postoperatively (Figure 3). Radiographic assessment of stem alignment (Table 6) demonstrated that, in the standard stem group, neutral alignment was most common (27 cases), followed by valgus (4 cases) and varus positioning (4 cases). In the short stem group, neutral alignment was observed in 24 cases, while valgus and varus alignment were identified in 4 and 7 cases, respectively.

Radiological assessment (Table 6) showed no statistically significant differences in stem alignment between the studied groups (*p* = 0.60). Neutral alignment was achieved in 68.6% of patients in the short-stem group and in 77.1% of patients in the standard-stem group. The effect size measured by Cramér’s V was 0.12, indicating a very weak (negligible) association between implant type and its radiological positioning. Although varus alignment was observed more frequently in the short-stem group (20.0% vs. 11.4%), this difference did not reach statistical significance.

The analysis indicates that stem positioning errors (varus/valgus malalignment) do not significantly compromise overall clinical outcomes, as reflected by preserved total HHS and WOMAC scores (Table 7). Although a nominal difference was observed in the postoperative WOMAC stiffness subscale favoring neutral alignment (median 1.0 vs. 2.0; nominal *p* = 0.039), this association did not retain statistical significance after adjusting for multiple comparisons (FDR-adj = 0.195).

The following comparison is descriptive (based on subgroup medians) rather than derived from a formal statistical interaction term, and should be interpreted accordingly. Exploratory subgroup observations regarding the combination of stem type and radiological positioning suggested that potential positional deviations might be slightly more pronounced in the short-stem group, particularly concerning patients’ subjective perception of postoperative stiffness. Conversely, the standard stem appeared less sensitive to alignment variations within this cohort. However, given the limited sample size of non-neutral alignments and the lack of significance after multiplicity adjustment, these descriptive trends remain exploratory and warrant cautious interpretation.

## 4. Discussion

Degenerative changes in the hip joint are one of the most common causes of musculoskeletal dysfunction, resulting in pain and reduced quality of life for patients. [42]. According to the World Health Organization, it is classified as a lifestyle disease, with approximately 40% of cases affecting the hip joint [43]. It is the second leading cause of patient referral to specialists, after spine conditions. Currently, the most commonly used and effective treatment for advanced degenerative changes is hip replacement surgery [17].

The presented study focused on comparing two stems of different lengths. The choice of femoral stem in clinical practice may be influenced by several patient- and anatomy-related factors, including femoral canal morphology, metaphyseal bone stock, bone quality, anticipated fixation, and the surgeon’s intraoperative assessment [44]. In the present cohort, stem selection was based on preoperative radiographic evaluation and intraoperative assessment.

The findings of this study demonstrate that both short-stem and standard-stem prostheses were associated with significant improvements in patient outcomes. The finding that both stem groups improved postoperatively is consistent with Lee et al. [45], who reported substantial functional improvement following both short-stem and standard-stem THA without demonstrating a clear functional superiority of either stem design. In their study, the mean HHS increased markedly from approximately 50 points preoperatively to over 90 points at final follow-up in both groups, while WOMAC score values decreased significantly from approximately 75 to around 23, indicating reduced pain and improved joint function, which aligns with our observations. It is worth noting that functional outcomes were assessed at 6 weeks after surgery, which represents a relatively early postoperative time point and may still reflect the ongoing recovery process. However, the substantial proportion of patients reported outcome results (PROM) improvement occurs during the early recovery period. Canfield et al. [46] found that 92.7% of total WOMAC improvement after THA was achieved by 6 months. These findings support the relevance of early postoperative assessment, but do not establish 6 weeks as a surrogate for longer-term functional outcomes.

An important distinction should therefore be made between the early functional assessment and the longer-term clinical follow-up. HHS and WOMAC were assessed in the present study only up to 6 weeks postoperatively, whereas the subsequent 3–6-year follow-up was used to monitor implant-related clinical outcomes, including stability, revisions, and complications. Consequently, the between-group differences observed in WOMAC scores reflect differences in early postoperative recovery and should not be interpreted as evidence of long-term functional superiority of either stem design. Whether these early differences persist, diminish, or disappear over longer follow-up cannot be determined from the present data.

The need for cautious interpretation of early functional differences is further illustrated by the randomized comparison of short- versus standard-stem prostheses, in which the HHS advantage observed for the short-stem group at 6 weeks and 3 months was no longer statistically significant at 6 or 12 months [47]. Therefore, further follow-up with repeated HHS and WOMAC assessments is warranted to determine whether the differences observed in the present study persist over time.

Interestingly, comparative analysis in this work of the two stem types revealed that patients receiving the short-stem prosthesis had significantly lower WOMAC total scores compared with those receiving the standard-stem prosthesis. These findings are also supported by evidence reported in other studies. The literature indicates that shorter stems can be an effective alternative to standard stems, provided proper implantation technique is used. Retrospective analyses demonstrate promising clinical outcomes with short stems, particularly in reducing pain in the proximal thigh. Briem et al. achieved good or excellent HHSs in 96% of cases after implantation of 155 short stems, with only one revision due to aseptic loosening [48]. Observational studies have reported favorable clinical outcomes with short-stem prostheses. However, these findings do not necessarily indicate superiority over conventional stems. Consistently, a systematic review and meta-analysis by Babu et al. [49] found no significant differences in HHS or WOMAC scores between short- and conventional-stem THA.

The results of the present study demonstrated significant differences in the total WOMAC score, as well as in the “function” subscale, in favor of the short-stem prosthesis.

These findings differ from those of several randomized controlled trials and meta-analyses, which have generally reported comparable functional outcomes, including HHS and WOMAC scores, between short-stem and conventional-stem THA, particularly at intermediate and longer-term follow-up [50,51,52]. The potential advantages of short stems reported in the literature appear to be more consistently associated with preservation of proximal bone stock, favorable bone remodeling, and reduced stress shielding rather than with superior overall functional outcomes.

Interestingly, a significant difference was also observed in the “stiffness” subscale, favoring the standard prosthesis. This finding may be considered somewhat unexpected, given that previous reports have indicated an association between larger stem sizes and increased postoperative stiffness [53]. One possible explanation is related to biomechanical reconstruction. In short-stem total hip arthroplasty, achieving optimal femoral offset and lateralization can be more technically demanding. Suboptimal restoration of these parameters may negatively affect the lever arm and function of the hip abductor muscles, potentially leading to their relative weakness. As a consequence, compensatory increases in tension within the joint capsule and surrounding soft tissues may occur, which could contribute to a subjective sensation of stiffness [42]. Statistical analyses in our work confirmed a significant association between WOMAC stiffness scores and prosthesis alignment, specifically between neutral and non-axial stem positioning. Patients with off-axis alignment reported greater postoperative stiffness compared with those with neutral positioning, thereby supporting the earlier hypothesis that malalignment contributes to increased subjective joint stiffness following surgery. However, because this between-group difference did not retain statistical significance after adjustment for multiple comparisons, this finding should be regarded as exploratory rather than confirmatory.

The study results demonstrated a change only in the WOMAC scores, whereas no statistically significant differences were observed in the HHS results. This discrepancy may be explained by the fact that the HHS combines clinician-assessed components with patient-reported measures of pain and function, while the WOMAC is a fully patient-reported outcome measure [54]. Similar findings were reported by Bertugli et al. [55], who, in a comparison of direct lateral versus posterior-lateral approaches in robotic-assisted total hip arthroplasty, observed significant differences in postoperative WOMAC scores between groups, whereas no such differences were detected using the HHS, indicating that the two instruments may yield divergent results within the same cohort. At the same time, it is important to emphasize that both scales are characterized by high reliability and validity in assessing outcomes following total hip arthroplasty and are widely used in clinical and research practice [54].

Complications leading to revision total hip arthroplasty account for a clinically relevant proportion of cases (on average 4% within 10 years) [56] and represent a significant threat to patient health and postoperative recovery. Numerous studies have addressed this issue in the context of stem design, particularly comparing standard and short stems. Molli et al. [57], in an analysis of 658 endoprostheses (389 standard and 269 short), found no significant differences in HHS, but reported a lower rate of intraoperative complications in the short-stem group (0.4% vs. 3.1% for standard stems). In our study, complications were observed in both groups: in the standard-stem group, one case of peroneal nerve neuropraxia and one periprosthetic fracture were noted, while in the short-stem group, a single dislocation occurred on postoperative day 11, with no further complications during follow-up. However, due to the small number of events, our study was not powered to detect differences in complication rates. These findings highlight the need for further research focusing on complication profiles in relation to stem design, which requires large patient cohorts to ensure adequate statistical power.

Several limitations of this study should be acknowledged. First, functional outcomes were assessed only preoperatively and at 6 weeks postoperatively. Thus, our findings reflect early postoperative recovery and do not establish whether the observed differences between short- and standard-stem groups persist at 6–12 months or during longer-term follow-up. Furthermore, the radiographic assessment was limited to postoperative stem positioning and alignment and did not include longitudinal evaluation of periprosthetic bone remodeling, stress shielding, or changes in bone mineral density. Therefore, no conclusions regarding differences in long-term bone remodeling between the two stem designs can be drawn from the present study.

Moreover, its retrospective, non-randomized design inherently carries a risk of selection bias and unmeasured confounding. Although multivariable adjustments and sensitivity analyses were performed to control for baseline differences, residual confounding cannot be entirely ruled out. In particular, several potentially relevant determinants of implant selection, including detailed femoral morphology, bone quality, metaphyseal bone stock, and other clinical or anatomical characteristics, were not systematically captured and therefore could not be included in the multivariable regression model. Although the model adjusted for age and BMI, residual confounding related to these unmeasured factors cannot be excluded. Accordingly, the observed association between stem type and postoperative functional outcome should be interpreted as an adjusted association rather than evidence of a causal effect of stem type. Second, the study is based on a relatively modest single-center cohort (*n* = 70), which limits the statistical power of subgroup analyses (such as radiographic malalignment evaluations) and precludes broad generalization. Consequently, findings regarding secondary clinical nuances should be regarded as exploratory. Future prospective, multi-center trials with larger patient populations and longer follow-up periods are warranted to confirm these observations and better establish the long-term clinical superiority of specific femoral stem designs.

## 5. Conclusions

Results presented in this work identified stem type as the only variable significantly and independently associated with superior functional outcome, with patients receiving the short-stem prosthesis demonstrating nearly sixfold higher odds of achieving better postoperative WOMAC results than those treated with the standard stem. In contrast, neither age nor BMI significantly influenced functional recovery. These findings indicate an association between stem type and early patient-reported functional outcome in the present cohort. However, given the retrospective design and the 6-week functional assessment, they should not be interpreted as evidence of a causal effect or sustained functional superiority of the short-stem design.

## Figures and Tables

**Table 1:** Baseline demographic characteristics in the short-stem and standard-stem study groups.

Parameter	Short-Stem Hip Prosthesis	Standard-Stem Hip Prosthesis	*p*-Value
Age [years], median (IQR)	67.0 (57.5–72.0)	67.0 (61.5–69.5)	0.855
Gender (F/M), *n* (%)	15/20 (42.9/57.1)	22/13 (62.9/37.1)	0.093
BMI [kg/m^2^], median (IQR)	27.72 (24.1–32.2)	28.04 (24.46–31.4)	0.747

Abbreviations: BMI, body mass index. Data are presented as medians and interquartile ranges (IQRs). Statistical significance was determined using the Mann–Whitney U test for continuous variables and the Chi-square test for categorical data (gender), with the level of significance set at *p* < 0.05 (indicated by an asterisk).

**Table 2:** Comparison of perioperative inflammatory markers and blood count parameters between the short-stem and standard-stem hip prosthesis groups.

Parameter	Time Point	Short-Stem Hip Prosthesis (*n* = 35)	Standard-Stem Hip Prosthesis (*n* = 35)	*p*-Value
CRP [mg/L], median (IQR)	Pre-op	0.2 (0.1–0.4)	0.4 (0.3–0.9)	**0.003** * (FDR-adj **0.018** *)
	Post-op	17.2 (12.7–22.8) †	19.8 (16.9–23.1) †	0.119 (FDR-adj 0.238)
HGB [g/dL], median (IQR)	Pre-op	13.3 (12.1–14.0)	13.2 (12.5–14.3)	0.782 (FDR-adj 0.782)
	Post-op	11.5 (10.1–12.2) †	11.1 (10.2–11.9) †	0.738 (FDR-adj 0.782)
LEU [109/L], median (IQR)	Pre-op	7.1 (6.2–8.3)	8.1 (7.0–9.5)	0.060 (FDR-adj 0.180)
	Post-op	10.2 (8.4–13.0) †	11.3 (9.9–13.8) †	0.137 (FDR-adj 0.238)

Data are expressed as medians and interquartile ranges (IQR: 25th–75th percentiles). Abbreviations used: CRP, C-reactive protein; HGB, hemoglobin; LEU, leukocytes; Pre-op, preoperative; Post-op, postoperative; FDR-adj, Benjamini–Hochberg false discovery rate adjusted *p*-value. The *p*-values in the final column indicate results of the Mann–Whitney U test for comparisons between the standard-stem and short-stem groups, along with Benjamini–Hochberg false discovery rate adjusted *p*-values (FDR-adj) where applicable. Bold values with an asterisk (*) denote statistical significance (*p* < 0.05). Statistically significant intra-group improvements from the preoperative to the postoperative period (*p* < 0.001 for all parameters), as determined by the Wilcoxon signed-rank test, are indicated by the dagger symbol (†).

**Table 3:** Preoperative and postoperative clinical outcomes according to HHS and WOMAC scores.

Clinical Parameter	Short-Stem Hip Prosthesis (*n* = 35)	Standard-Stem Hip Prosthesis (*n* = 35)	*p*-Value	Effect Size (r)
HHS, median (IQR):				
Pre-op	52.0 (43.5–59.5)	48.0 (38.0–57.0)	0.259 (FDR-adj 0.370)	-
Post-op	91.0 (89.0–95.0) †	91.0 (87.5–94.0) †	0.426 (FDR-adj 0.491)	-
WOMAC Total Score, median (IQR)				
Pre-op	60.0 (57.0–65.0)	63.0 (60.0–68.0)	0.065 (FDR-adj 0.120)	-
Post-op	17.0 (13.0–18.5) †	22.0 (17.5–24.0) †	6.4 × 10^−5^ * (FDR-adj < 0.001 *)	**0.45 (Medium/Large)**
WOMAC Subscales, median (IQR):				
Pain Pre-op	13.0 (11.5–14.0)	13.0 (11.5–15.0)	0.491 (FDR-adj 0.491)	-
Post-op	1.0 (1.0–2.0) †	2.0 (1.0–3.0) †	0.056 (FDR-adj 0.120)	-
Stiffness Pre-op	4.0 (3.0–5.0)	4.0 (3.0–5.0)	0.482 (FDR-adj 0.491)	-
Post-op	2.0 (1.0–3.0) †	1.0 (1.0–2.0) †	0.013 * (FDR-adj 0.043)	**0.26 (Small)**
Function Pre-op	43.0 (40.0–47.5)	48.0 (42.0–51.5)	0.072 (FDR-adj 0.120)	-
Post-op	11.0 (8.0–16.0) †	19.0 (15.5–20.5) †	1.7 × 10^−12^ * (FDR-adj < 0.001 *)	**0.62 (Large)**

Data are presented as medians and interquartile ranges (IQR). Abbreviations: HHS, Harris Hip Score; WOMAC, Western Ontario and McMaster Universities Osteoarthritis Index; Pre-op, preoperative; Post-op, postoperative; FDR-adj, Benjamini–Hochberg false discovery rate adjusted *p*-value. For WOMAC scores, lower values represent superior clinical outcomes. The *p*-values in the final column represent the results of the Mann–Whitney U test for between-group comparisons, along with Benjamini–Hochberg false discovery rate adjusted *p*-values (FDR-adj) where applicable. Bold values with an asterisk (*) indicate statistical significance at *p* < 0.05. The dagger symbol (†) denotes a statistically significant improvement from the preoperative to the postoperative period within each group (*p* < 0.001), as determined by the Wilcoxon signed-rank test.

**Table 4:** Spearman’s rank correlation analysis between patient characteristics and postoperative WOMAC scores.

Variable	Correlation Coefficient (Rs)	*p*-Value	Interpretation
Age vs. WOMAC Total	0.033	0.787	Negligible correlation (Insignificant)
BMI vs. WOMAC Total	0.103	0.395	Weak correlation (Insignificant)
Age vs. WOMAC Function	0.012	0.921	Negligible correlation (Insignificant)
BMI vs. WOMAC Function	0.098	0.419	Weak correlation (Insignificant)

Abbreviations: Rs, Spearman’s rho correlation coefficient; BMI, Body Mass Index; WOMAC, Western Ontario and McMaster Universities Osteoarthritis Index. Statistical significance defined as *p* < 0.05.

**Table 5:** Multivariable logistic regression analysis of factors associated with superior functional outcome (WOMAC score).

Variable	Odds Ratio (OR)	95% Confidence Interval (CI)	*p*-Value
Stem Type (Short-stem = 1 Standard-stem = 0)	5.87	2.05–16.80	0.001 *
Age [years]	1.01	0.94–1.08	0.787
BMI [kg/m^2^]	1.04	0.95–1.15	0.395

Abbreviations: OR, odds ratio; CI, confidence interval; BMI, body mass index. Statistical significance is indicated by an asterisk (*) where *p* < 0.05. The multivariable logistic regression model was used to identify factors associated with a superior functional outcome (defined as a postoperative WOMAC score at or below the median of 18 points).

**Table 6:** Comparison of short-stem and standard-stem alignment. Radiographic evaluation of femoral stem alignment in the short-stem and standard-stem groups.

Stem Alignment	Short-Stem Hip Prosthesis (*n* = 35)	Standard-Stem Hip Prosthesis (*n* = 35)	*p*-Value
Neutral alignment	24 (68.6%)	27 (77.1%)	0.602
Valgus alignment	4 (11.4%)	4 (11.4%)	
Varus alignment	7 (20.0%)	4 (11.4%)	

Data are presented as absolute numbers and percentages, *n* (%). The distribution of stem alignment was compared between groups using Fisher’s exact test. Effect size was calculated using Cramer’s V coefficient. (Cramer’s V = 0.12). Statistical significance defined as *p* < 0.05.

**Table 7:** Comparison of postoperative clinical parameters based on radiographic stem alignment.

Clinical Parameter	Neutral Alignment (*n* = 51)	Non-Neutral Alignment (*n* = 19)	*p*-Value
HHS total score	94.5 (89.0–100.0)	92.0 (88.0–98.0)	0.412 (FDR-adj 0.412)
WOMAC total score	12.0 (8.0–18.0)	16.0 (10.0–24.0)	0.154 (FDR-adj 0.352)
WOMAC Pain	2.0 (1.0–4.0)	3.0 (2.0–5.0)	0.287 (FDR-adj 0.359)
WOMAC Stiffness	1.0 (0.0–2.0)	2.0 (1.0–4.0)	0.039 * (FDR-adj 0.195)
WOMAC Function	8.0 (6.0–12.0)	10.0 (8.0–15.0)	0.211 (FDR-adj 0.352)

Data are presented as medians and interquartile ranges (IQR). Abbreviations: HHS, Harris Hip Score; WOMAC, Western Ontario and McMaster Universities Osteoarthritis Index; FDR-adj, Benjamini–Hochberg false discovery rate adjusted *p*-value. For WOMAC scores, lower values represent superior clinical outcomes. The *p*-values in the final column represent the results of the Mann–Whitney U test for between-group comparisons (Neutral vs. Non-neutral alignment) along with Benjamini–Hochberg false discovery rate adjusted *p*-values (FDR-adj) where applicable. Bold values with an asterisk (*) indicate statistical significance at *p* < 0.05.

## Data Availability

The datasets generated and analyzed during the current study are available at the University of Kalisz, Plac Wojciecha Bogusławskiego 2, 62-800 Kalisz, Poland, and may be obtained from the corresponding authors upon reasonable request.
